# Efficacy of various core decompression techniques versus non-operative treatment for osteonecrosis of the femoral head: a systemic review and network meta-analysis of randomized controlled trials

**DOI:** 10.1186/s12891-021-04808-2

**Published:** 2021-11-15

**Authors:** Quanzhe Liu, Wenlai Guo, Rui Li, Jae Hyup Lee

**Affiliations:** 1 Department of Orthopedic Surgery, Seoul National University, College of Medicine, Seoul, South Korea; 2 Department of Hand Surgery, The Second Hospital of Jilin University, Changchun, Jilin Province China; 3 Department of Orthopedic Surgery, Seoul Metropolitan Government - Seoul National University Boramae Medical Center, 20 Boramaero 5-gil, Dongjak-gu, Seoul, 07061 South Korea

**Keywords:** Osteonecrosis of the femoral head (ONFH), Orthopedic procedures, Joint-preserving therapy (JPT), Total hip arthroplasty (THA), Radiographic progression, Harris hip score (HHS)

## Abstract

**Background:**

Various Joint-preserving therapy (JPT) methods have been performed and tried in recent decades, but their results and efficacy were inconsistent and controversial. The purpose of this study is to evaluate its effectiveness and whether there are statistical differences in treatment between different interventions based on published RCT studies.

**Methods:**

Following the PRISMA-NMA checklist, Medline, EMBASE, Web of Science, and Cochrane Library databases were searched and collected related RCT studies. The sources were searched from inception up to October 30, 2020. The primary outcomes including the rate of radiographic progression and conversion to THA and the secondary outcome -Harris Hip Scores (HHS) were extracted and compared in a Network meta-analysis.

**Results:**

Seventeen RCT studies involving 784 patients (918 hips) with seven interventions including CD (core decompression), CD + BG (bone graft), CD + TI (tantalum rod implantation), CD + CT (Cell therapy), CD + BG + CT, VBG (vascularized bone graft), and nonsurgical or conservative treatment for ONFH were evaluated. In the radiographic progression results, CD + CT showed a relatively better result than CD, CD + BG and non-surgical treatment, the surface under the cumulative ranking curve (SUCRA) plot displayed that CD + CT (96.4%) was the best, followed by CD (64.1%).In conversion to THA results, there were no significant differences between the JPT methods and non-surgical treatment. In HHS, there was also no significant difference, other than CD + BG showed a statistical difference than non-surgical treatment only in terms of Cis, but the SUCRA was highest in non-surgical treatment (80.5%) followed by CD + CT (72.8%).

**Conclusions:**

This Net-work meta-analysis demonstrated that there was no statistical difference in the outcome of radiographic progression and conversion to THA, also in HHS, other than CD + CT showed a relatively superior result in radiographic progression than nonsurgical treatment, namely, it’s maybe an effective method for delaying disease progression or reducing disease development based on current evidence.

**Supplementary Information:**

The online version contains supplementary material available at 10.1186/s12891-021-04808-2.

## Background

Osteonecrosis of the femoral head (ONFH) is a common but refractory disease in the orthopedics field. The initial manifestations are localized, partial necrosis, but with the development of advanced hip joint disease, it may progress to complete elimination of femoral head.

The annual incidence in the USA was estimated to be between approximately 10,000 and 20,000 cases [1]. and 0.01% in the German-speaking countries, with 5000 to 7000 persons affected [2]. If there is no timely and effective treatment in the early stage, 80% of cases will have femoral head collapse and end-stage degenerative joint disease within 2 years, and eventually need to total hip arthroplasty (THA) [3–5]. The THA method has good function improvement and pain relief [6–8], but its application in young patients faces some problems. For example, the prosthetic replacements rarely last for a lifetime and bring a huge financial burden increasing the overall cost of managing this condition due to the requirement for revision THA [9, 10] between ages of 30–50 years considered to be the most susceptible [5, 11, 12]. The principles for considering ONFH treatment include the termination of pathologic progression and the restoring of weight-bearing capacity, many different joint-preserving therapy (JPT) methods have been performed in recent decades-years. Based on published data in the USA by Sodhi, N [13], although there was an increasing rate of THA for ONFH (75 to 88%) [14] during 1992 to 2008, decreased during 2009 to 2015 and found that the rate of joint-preserving procedures showed a trend of relative growth, especially in patients aged <50 years.

Until now, various joint-preserving therapies (JPT) have been further explored with promising results, including core decompression (CD), Extracorporeal shockwave therapy,

vascularized and non-vascularized bone graft, CD combined with bone mesenchymal stem cells (BMSC) or other bone marrow cells and applications of biomaterials like tantalum implantation et al. before the femoral head collapse. Each has its advantages, core decompression can decrease intraosseous pressure, alleviate bone marrow edema and improve blood supply for the femoral head [15–17], mesenchymal stem cells (MSCs) can release exosomes that contain cytokines promoting osteogenesis, chondrogenesis, and angiogenesis [18, 19]. Bone graft allows for subchondral bone remodeling and healing [20, 21] even tantalum rod implantation has become a good choice of mechanical substitute because of its superior strength, fatigue properties, biocompatibility [22]. And vascularized bone graft (VBG) provides structural support, but also restores vascular supply to enhance lesion healing [23–25]. Despite this, the results of treatment effect have been reported inconsistently from published studies and there was a lack of consensus as to which methods were more effective or superior.

Given the continuing uncertainties, this study, therefore, was designed as a systemic review and network meta-analysis to assess the effectiveness between various joint-preserving procedures and non-surgical conservative treatment for ONFH using an updated systematic review and meta-analysis including all relevant RCTs published to date to provide the selection and application of clinical treatment.

## Methods

### Protocol and registration

This systemic review and Network Meta-analysis (NMA) were conducted following the Preferred Reporting Items for Systemic Reviews and Meta-analyses for Network Meta-analysis (PRISMA-NMA) reporting guideline [26]. The protocol has been registered in PROSPERO with the registration No: CRD42020214489.

### Search strategy

Ovid Medline, EMBASE, Web of Science, and Cochrane Library databases were searched to collect all published evidence from inception up to and including Oct 30, 2020. Search terms included extensive controlled vocabulary in various combinations, supplemented with keywords including ‘Femur Head’, ‘Joints’, ‘Osteonecrosis’, ‘Necrosis’, ‘Orthopedic Procedures’. The search strategy used in Medline via OVID is presented in Appendix 1. Besides, the reference reports of previous systematic reviews, meta-analyses, and randomized controlled trials (RCTs) were manually reviewed.

### Eligibility criteria

Only RCTs were included. The PICO information was as follows: patients(P): the patients who were diagnosed as ONFH and more than 18 years age; intervention(I): various joint-preserving procedures including non-vascular or vascular bone graft, tantalum implantation, cell-therapy (CT) including Mesenchymal stem cells, bone marrow aspirate concentrate, bone marrow mononuclear cells, et al. and non-surgical or physiotherapy treatment; Comparison(C): different types of treatment as a direct or indirect comparison; Outcome measures(O): the primary outcome were the rate of conversion to total hip replacement arthroplasty (THA); the rate of radiographic progressions to next stage; the secondary outcome was HHS (Harris Hip Score) to assess functional recovery; The study design (SD): RCT. The language was limited to English.

The exclusion criteria were as follows: (1) non-RCT, laboratory scientific or other non-relevant studies; (2) the study shared the same data set; (3) the study combined drug therapy effect with interventions or osteotomy. (4) the study focused on undesired outcomes or interventions. (5) literature report could not be extracted or converted into valid data.

### Data extraction and outcome assessment

The following information was extracted independently: the first author; publication year; sample size; the number of hips; type of intervention; sex ratio; age; stage (ARCO or Ficat or Steinberg); risk factors and follow-up time. All treatments using cell extraction in ONFH treatment are classified as cell therapy (CT) owing to no uniform standard for cell extraction and classified vascular and avascular bone graft as VBG and BG according to whether vessels were used to supply blood. And non-surgical treatment includes a variety of physical and rehabilitation training such as physical shockwave therapy. The risk factors and clinical stages were also extracted and analyzed for statistical differences, because they maybe a potential interference factor and affect the results.

Clinical primary outcomes containing the number of conversions to THA and radiographic progression to the next stage. The secondary outcome of interest was the HHS (Harris Hip Score) to assess clinic function (pain, joint activity, absence of deformity, and range of motion). The maximum score is 100 and higher indicates a better treatment result. Because the postoperative follow-up time was different, all the data results were based on the last follow-up reported outcomes of each study.

### Risk of Bias assessment

The Cochrane Collaboration’s risk of bias tool [27] was used to assess bias according to Review Manager (version 5.3). The disagreements were resolved by the third reviewer and the methodology for each study was graded as ‘high’, ‘low’, or ‘unclear’ reflecting the risk of bias.

### Statistical analysis

A multiple treatment comparison NMA was performed under a frequentist framework using a random-effects model. The network and mvmeta packages in Stata statistical software version 14.0 MP (State Corp) [28, 29] were used to analyze the data. The network plots were used to summarized geometry of the evidence network indicate the type of various interventions, the number of patients, and the amount of pair-wise comparison. The consistency of the network was checked with local and global inconsistency tests. Each closed loop in the network was assessed to confirm local inconsistency between direct and indirect effect estimates and only triangular (formed by three treatments all compared with one another) loops were considered.

The summary of mean differences and 95% confidence intervals (Cis) together with their predictive intervals (PrIs) were presented between comparisons. PrIs provide an interval within which the estimate of a future study is expected to be.

The surface under the cumulative ranking curve (SUCRA) is a relative ranking measure that accounts both for the location and the variance of all relative treatment effects [30]. A lower SUCRA value was regarded as a better result for the primary outcome and a higher SUCRA value was regarded as a better result for the secondary outcome. A comparison-adjusted funnel plot was used to assess the presence of small-study effects [31].

## Results

### Baseline characteristics of included studies

**Fig. 1:**
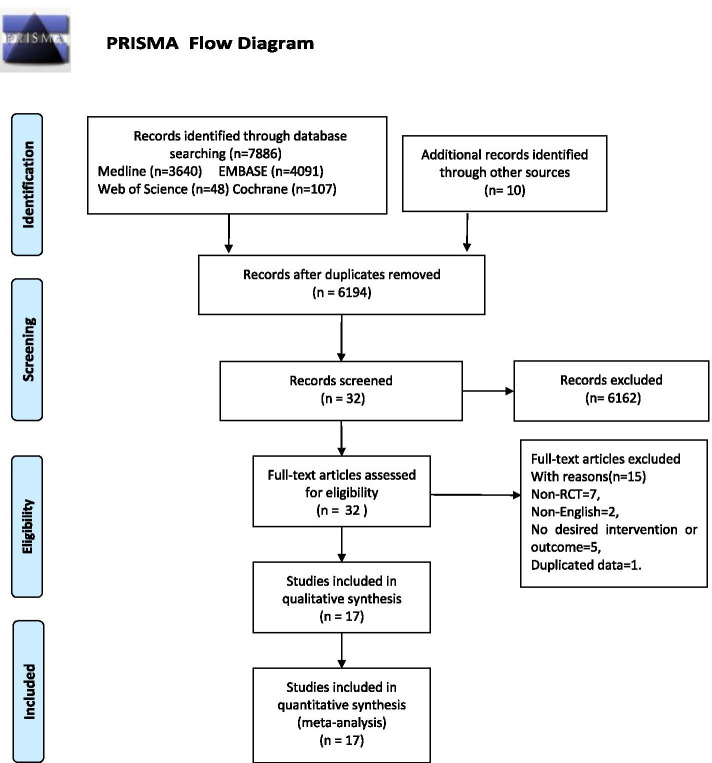
PRISMA flow diagram details the process of relevant clinical study selection

A PRISMA diagram summarizes the literature search results and study selection for this systemic review and NMA is shown in Fig. 1. Of the 7896 citations identified through our literature search (1702 after duplicates were removed), 6162 were deemed ineligible after the title and abstract screening, leaving 32 to articles search for full-text review. In total, 17 RCTs [15, 17, 32–47] involving 784 patients (918 hips) met the inclusion criteria and were accepted. Including non-surgical treatment, Core decompression (CD), CD + bone graft (BG), CD + TI (tantalum rod implantation), CD + Cell therapy (CT), CD + BG + CT and vascularized bone graft (VBG). The basic characteristics of the included studies are summarized in Table 1.

**Table 1:** Basic characteristics of the included studies

No.	Author	Year	Comparisons (Intervention)	No.P	No.Hip	Sex(M/F)	Age	Stage(I-IV)	Risk factors				Follow-up
								ARCO	Steroid	Alcohol	Idiopathic	others	
**1**	Peng, K.	2020	CD + BG	30	38	17:13	46.7 ± 13.9	11:19:0:0	16	8	0	6	1 years
			CD + TI	30	38	15:15		13:17:0:0	17	6	0	7	
**2**	Hauzeur, J. P	2018	CD	19	23	13:6	49.7 ± 3.2	0:0:23:0	13	7	3	0	2 years
			CD + CT	19	23	14:5	48.0 ± 2.8	0:0:23:0	12	8	1	2	
**3**	Cao, L.	2017	CD + BG	21	21	16:5	31 ± 6	3:13:5:0	7	8	6	0	3 years
			VBG		21			2:13:6:0					
**4**	Pepke, W.	2016	CD	24	14	8:6	44.5 ± 3.3	0:14:0:0	NR				2 years
			CD + CT		11	6:5	44.3 ± 3.4	0:11:0:0					
**5**	Tabatabaee, R. M.	2015	CD	13	14	10:4	26.8 ± 5.8	2:7:5:0	9	0	5	0	2 years
			CD + CT	14	14	9:5	31 ± 11.4	3:9:2:0	10	0	4	0	
**6**	Zhao, D.	2012	CD	50	51	26:24	33.8 ± 7.70	2:49:0:0	13	7	13	17	5 years
			CD + CT	50	53	27:23	32.7 ± 10.5	3:48:0:0	10	11	16	13	
**7**	Wang	2005	Non-surg	23	29	20:3	39.8 ± 12.1	3:10:16:0	2	16	0	5	2 years
			CD + BG	25	28	23:2	39.9 ± 9.3	2:17:9:0	2	16	0	7	
**8**	Gangji	2011	CD	19	11	10:9	45.7 ± 2.8	2:9:0:0	9	1	1	0	5 years
			CD + CT		13		42.2 ± 2.6	2:11:0:0	11	1	1	0	
**9**	Sen, R. K.	2012	CD	40	25	18:7	65.7 ± 15.2	stage(I or II)	20	8	2	21	2 years
			CD + CT		26	19:7	66.2 ± 13.0						
								**Ficat**					
**10**	Ma, Y.	2014	CD + BG	18	24	13:5	35 ± 9.8	4:15:5:0	13	3	6	0	2 years
			CD + BG + CT	21	25	15:6		3:17:5:0	13	4	6	0	
**11**	Li, MY	2020	CD + BG	14	20	10:4	38.2 ± 8.1	0:11:9:0	9	5	6	0	10 years
			CD + BG + CT	17	21	12:5	34.1 ± 8.0	0:11:10:0	10	6	5	0	
**12**	Stulberg BN,	1991	CD	19	28	NR	38.6	18:14:21:0	10	3	4	2	18 month
			Non-surg	17	25				8	8	1	0	
**13**	Deqiang Li,	2016	CD + BG	20	23	27:12	36.5 (23–59)	21:26:0:0	8	23	0	8	2.5 years
			VBG	19	24								
								**Steinberg**					
**14**	Neumayr LD,	2006	CD	17	17	8:9	24.67	2:5:10:0	NR				3 years
			Non-surg	21	21	11:10	26.41	8:6:7:0					
**15**	Miao H	2015	CD + TI	30	36	12:18	32.6 ± 6.3	16:20:0:0	22	4	7	0	18 month
			CD	30	34	13:17	35.2 ± 5.8	14:20:0:0	24	5	8	0	
**16**	Koo KH	1995	CD + BG	17	18	16:1	45	10:7:1:0	2	14	2	0	2 years
			Non-surg	17	19	16:1	48	12:4:3:0	2	17	0	0	
**17**	Hu, B. J.	2018	CD	65	65	44:21	40.38 ± 6.63	NR	NR				4 years
			CD + BG	65	65	46:19	40.83 ± 6.73						

### Risk of bias of included researches

**Fig. 2:**
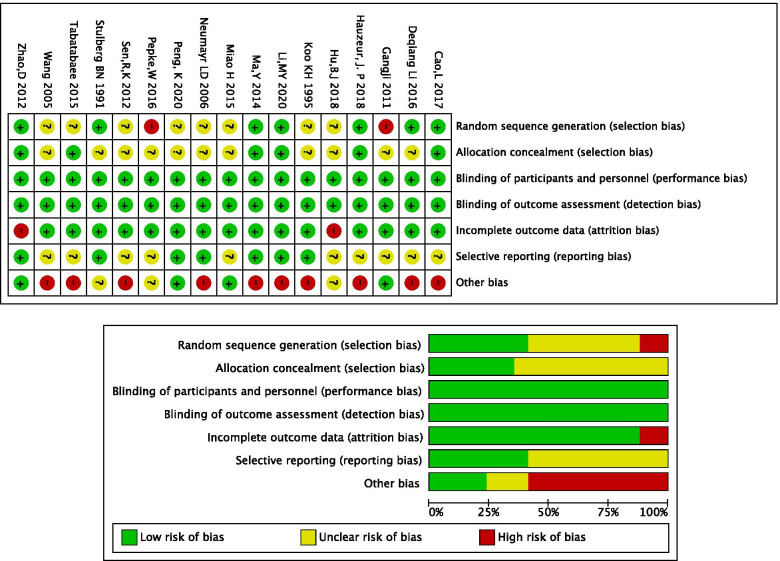
Details about the risk of bias assessment

The risk of bias of the included studies in this NMA was generally unclear or high. Overall, studies fulfilled the criteria for a judgment of a high risk of bias. Details about the risk of bias assessment were graphically summarized in Fig. 2. The study was deemed high risk.

### Results of meta-analysis

**Fig. 3:**
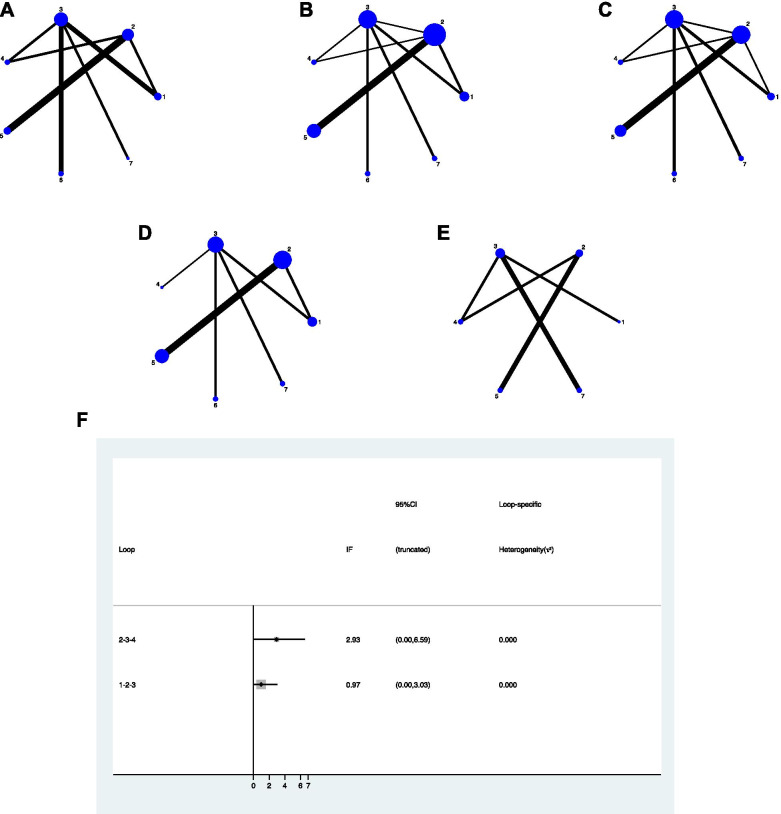
Network plot of the direct comparisons of the outcomes for all included studies. The width of the lines is proportional to the number of trials comparing every pair of treatments, and the size of every node is proportional to the number of randomized participants (sample size). **A**) risk factor of ONFH, **B**) stage 3 **C**) radiographic progression **D**) conversion to THA, **E**) HHS, **F**) the inconsistency plots of the direct and indirect comparisons, in radiographic progression

**Fig. 4:**
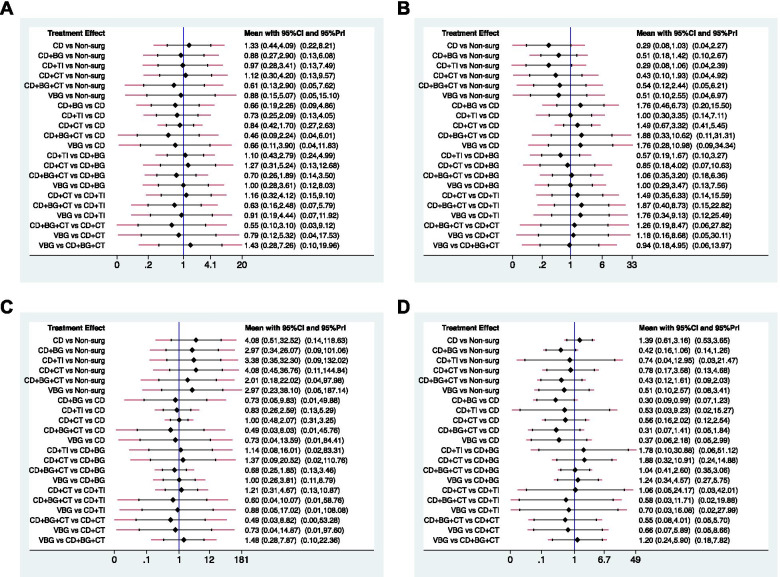
Confidence intervals and predictive intervals of the estimates of outcomes of risk factor of ONFH and stage 3. The black part corresponds to the 95% confidence interval and the red part to the 95% predictive interval. **A**) Steroid. **B**) Alcohol. **C**) Idiopathic. **D**) ONFH stage 3

Figure 3 A and B showed the network plot of the risk factors induced to ONFH and the proportion of stage 3 in each intervention, respectively. And the results showed that there was no significant statistical difference between each intervention in Figure 4.

#### Radiographic progression

Figure 3.C depicted the network plot of the various JPT methods comparing the rate of radiographic progression results. Seven interventions (CD, CD + BG, CD + TI, CD + CT, CD + BG + CT,VBG, and non-surgical treatment) were compared in 15 studies [15, 17, 32–36, 38, 39, 41–44, 46, 47] and pooled results. CD and CD + CT were compared directly more than the other treatment. CD and CD + CT was the most frequent comparator in our studies. Three comparisons (CD + BG vs CD + BG + CT, CD + BG vs VBG and CD vs CD + CT) were conducted using direct evidence alone. Five comparisons were performed using mixed evidence (both direct and indirect evidence) and 13 comparisons using indirect evidence alone.

**Fig. 5:**
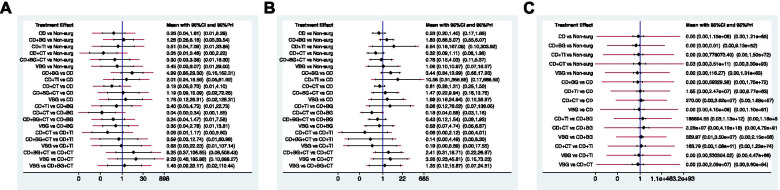
Confidence intervals and predictive intervals of the estimates of outcomes. The black part corresponds to the 95% confidence interval and the red part to the 95% predictive interval. **A**) the rate of radiographic progression. **B**) the rate of conversion to THA. **C**) HHS

**Fig. 6:**
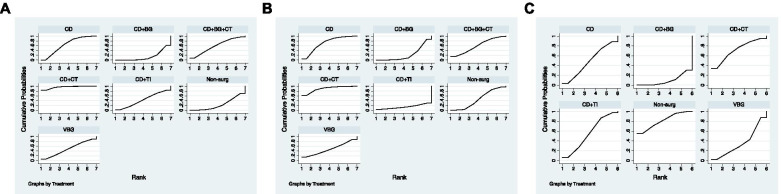
the Cumulative ranking curve of the outcomes of each JPT method. **A**) the rate of radiographic progression. **B**) the rate of conversion to THA. **C**) HHS

**Fig. 7:**
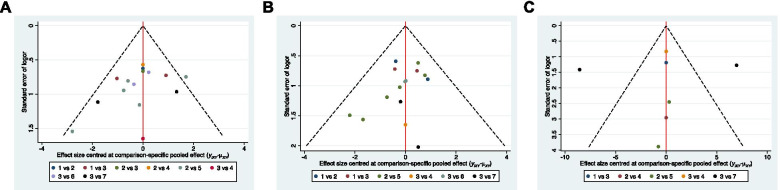
Comparison-adjusted funnel plots. **A**) the rate of radiographic progression. **B**) the rate of conversion to THA. C) HHS. 1: Non-surgical treatment. 2: Core decompression (CD). 3: CD + BG. 4: CD + TI. 5: CD + CT. 6: CD + BG + CT. 7: VBG

There were two closed loops and no significance in the local inconsistency between the direct and indirect point estimates (Fig. 3F), and no network inconsistency [χ^2^ (2) = 1.87, *p* = 0.392]. CD + CT showed a statistical difference and relatively superior result than non-surgical treatment, CD, and CD + BG, which were significant only in 95% Cis but not in 95% PrIs (Fig. 5A) which means that any future RCT could change the significance of the efficacy of these comparisons.

The cumulative ranking plot was drawn, and the SUCRA probabilities were calculated (Fig. 6A). According to the SUCRA value, CD + CT (96.4%) showed the relatively best result, followed by CD (64.1%), CD + BG + CT (59.2%), VBG (48.5%), CD + TI (43.3%), non-surgical treatment (24.3%) and CD + BG (14.3%). The comparison-adjusted funnel plots showed that the funnel plots were symmetrical around the zero lines, which suggested a less likely publication bias (Fig. 7A).

#### Conversion to THA

Figure 3D displayed the network graph of the seven interventions (CD, CD + BG, CD + TI, CD + CT, CD + BG + CT,VBG, and non-surgical treatment) that compared in 15 studies [15, 17, 32, 33, 35–39, 42–47] in terms of conversion to THA. CD and CD + CT were more studies when compared directly than the other JPT methods. Four comparisons (CD vs CT, CD + BG vs CD + TI, CD + BG vs CD + BG + CT, and CD + BG vs VBG) were conducted using direct evidence alone. Two comparisons were using mixed evidence and 15 comparisons using indirect evidence alone.

There was no available loop formed by the study arms, and loop-specific tests were not performed. No significant differences among the JPT methods in terms of both CIs and PrIs were found, other than CD + CT showed a lower rate than CD + BG only in terms of the Cis (Fig. 5B).

The SUCRA plots (Fig. 6B) showed that the rate of conversion to THA was lowest in CD + CT (90.4%), followed by CD (69.1%), CD + BG + CT (58%), VBG (47.6%), non-surgical (46.2%), CD + BG (24%), and CD + TI (14.8%). The comparison-adjusted funnel plots suggested a less likely publication bias (Fig. 7B).

### Harris hip scores

The network plot comparing the HHS was depicted in Fig. 3E. Six JPT methods (CD, CD + BG, CD + TI, CD + BG + CT, VBG and non-surgical treatment) were compared in 7 studies [17, 32, 36–38, 41, 46]. Five comparisons were conducted using mixed evidence and 10 comparisons using the indirect evidence alone. There was no available loop formed by the study arms.

There was no significant difference among these methods in CIs and PrIs other than CD + BG showed a higher score than non-surgical treatment only in terms of Cis (Fig. 5C). The SUCRA plot displayed that the HHS (Fig. 6C) was the highest in non-surgical treatment (80.5%), CD + CT (72.8%), CD + TI (54.0%), CD (48.7%), VBG (34.8%) and CD + BG (9.2%). Publication bias was less likely in the comparison-adjusted funnel plot (Fig. 7C).

## Discussion

. In recent years, many studies [15, 17, 32–47] have been made and various JPT methods have been carried out, but it remains challenges and controversies about efficacy. In our NMA, there was no statistical difference in radiographic progression and conversion to THA, also in HHS between JPT methods and non-surgical treatment, other than CD + CT showed a relatively superior result in radiographic progression.

Based on reported data in USA, THA was still the most commonly performed procedure for AVN and the annual percentage of patients managed using a THA accounted for 89.5% between 2009 and 2015, reported by Sodhi [13]. However, the success of non-replacement procedures is also critical to the patients’ quality of life and the impact on the healthcare system.

Simple core decompression (CD) has become the reference technique widely used in patients with early-stage ONFH Since popularized by Hungerford [48]. But the results of CD are always debated and controversial [11, 47, 49]. The efficacy of CD has been considered that it can decrease intraosseous pressure, alleviate bone marrow edema and improve blood supply for femoral head [15–17]. However, other studies [50–52] questioned and reported that, in fact, CD was not superior to non-surgical treatment, it cannot repair the femoral head which even lowers its biological strength and causes collapse. They found that when there is a subchondral fracture (47% in ARCO stage 3), compared with the pre-collapse stages (85% in ARCO stage 1 and 65% in ARCO stage 2), the success rate of core decompression is even worse [53, 54]. Koo KH at their RCT study also found that CD may be effective in symptomatic relief, but is of no greater value than conservative management in preventing collapse. Based on our NMA, there were no statistical differences in primary and secondary outcomes compared with other JPT methods and non-surgical treatment, although inferior to the CD + CT method in terms of radiographic progression.

Owing to CD’s unpredictable and different results especially in long-term results, and to improve the limitations, several other JPT methods have been proposed and improved in recent years and achieved some promising results on ONFH outcomes versus CD.

Cell therapy, especially stem cells, has been a research hotspot in recent years, and is used for tissue regeneration due to its ability to differentiate into multiple cell lineages [55]. It is generally believed that mesenchymal stem cells (MSCs) can release exosomes, which contain cytokines that promote bone formation, cartilage formation and angiogenesis, including bone morphogenetic protein 2, vascular endothelial growth factor and transforming growth factor β [18, 19]. Therefore, stem cell transplantation is expected to become a new method of ONFH combined with core decompression. The purpose of adding MSCs to the core decompression tunnel is to provide osteoprogenitor cells and vascular progenitor cells in the decompressed necrotic bone area to promote tissue regeneration and repair reported by Goodman et al. [56]. Since the CD + BMSCs for ONFH was proposed by Hernigou [57] et al. in 2006, many studies achieved their promising results. Gangji [44] et al. concluded that cell implantation delayed the progression of stage 1–2 osteonecrosis and decreased hip pain and joint symptoms more effectively than CD during sixty-month follow-up period. And at meta-analysis also found that it had better pain relief, clinical outcomes and provided a significant improvement in terms of survivorship over time compared with CD alone [58, 59].

However, there were also different results. Hauzer [35] at a double blind RCT comparing the CD with the CD + BMAC and found that implantation of it after CD did not produce any improvement of the evolution of ONFH in stage 3. Lim [60] et al. also assessed the clinical effects and radiological results according to a controlled trail and found that CD + CT method was ineffective in stage III–IV patients and there were no statistically significant differences between CD + CT and CD + BG in success rate or the clinical and radiographic results. In our NMA, we found that there was statistical difference and had relatively superior result than non-surgical treatment, CD, CD + BG and VBG in radiographic progression, but no in conversion to THA and HHS. Therefore, we think that it may be an effective method for delaying disease progression or reducing disease development based on current evidence especially in stage I and II.

Non-vascularized bone graft was also a choice for ONFH, Since Phemister [61] as one of first authors described the use of non-vascularized bone graft (NVBG) from tibia for ONFH. Multiple studies have shown that the success of this support following necrotic segment decompression to allow for subchondral bone remodeling and healing [20, 21]. Deqiang [38] et al. at RCT study reported that CD + BG can relieve hip pain, improve function with much lesser surgical trauma compared to VBG, so it is a better choice for ONFH. However, Wang C [46] reported that the shock-wave conservative treatment appeared to be more effective than CD + BG in early-stage ONFH, although the mechanism remains unknown. Based on our network results and rankings results, CD + BG had no obvious advantage in terms of primary and secondary outcomes and no statistically differences.

As a good choice of mechanical substitute, the technique of porous tantalum rod implantation (TI) was applied in medical science more than half a century [32], Because of its advantages of superior strength, fatigue properties, biocompatibility and initial stability for bones to those of natural osseous grafts, and they have low cytotoxicity and bacterial adhesion force [22]. Hua KC [62] at their meta-analysis showed that CD combined with TI showed satisfactory clinical results. However, some research argues that this method can only provide temporary structural support until new bone ingrowth in the necrotic lesion and the absence of new bone tissue growing into porous tantalum rod in necrotic lesions made this method less ideal [63, 64]. According to histopathologic search and analysis, the clinically failed implants found that among the 15 specimens, there were 14 cases of residual osteonecrosis, and all cases had subchondral fractures of the femoral head, among which 60% of the femoral head collapsed by Tanzer M [64]. Based on our study, CD + TI did not have significantly advantage as compared with other JPT methods in primary and secondary outcomes and no statistically difference.

VBG is also one of the popularized JPT methods for ONFH, some studies had showed that it was a better treatment option than CD combined with non-vascularized bone graft because not only provides structural support, but also restores vascular supply to enhance lesion healing [23–25]. However, high technical requirements and a relatively low success rate of surgery have to be considered. As reported, the failure rate of vascularized grafts ranging from 4 to 30% [65, 66] and most of them were found in chronic steroid users as reported in the literatures [67]. Meloni et al. [68] reported that vascularized fibular graft (VFG) failure appears to be related to the negative effect of creeping substitution and supports unbalanced bone resorption enhanced by corticosteroids. Although Ji Wang et al. [69] showed that VFG was superior effect on reducing treatment failure rates at their network meta-analysis, our results was not exactly consistent with them, the effect was not so superior compared other treatment. Although the risk factors for ONFH were not statistically different in each intervention, we found that chronic steroid-using is one of the main induce factor to ONFH and accounted for 25% (15/60) in the VBG group. This may be one of the main reasons for the difference. Reviewing the literature in our studies, VBG may be an effective treatment, but based on our results, considering various factors, VBG was not the best option treatment for ONFH especially in chronic steroid users.

There are several limitations in our NMA. First, this study did not make further analysis and summary according to the size and location of the necrotic lesion. Second, the adjuvant procedures and the follow-up time within each category were hard to be consistent. Third, the number of subjects was still limited and a larger scale RCT study will be necessary to confirm whether various joint-preserving procedures prevents the disease progression or are effective in ONFH. Fourth, the risk of bias of the included studies in this NMA was generally unclear or high, higher quality RCT research will be needed in the future.

## Conclusion

This network meta-analysis assessed the various JPT methods containing the conservative non-surgical treatment in patients of ONFH. Although all available JPT methods have not been evaluated and further studies are needed, our NMA provides some important information about various methods of Joint-preserving treatment and references to select appropriate JPT methods in ONFH. The data suggest that there was no statistically difference in radiographic progression and conversion to THA, also in HHS between above JPT methods, other than CD + CT showed a relatively superior result in radiographic progression than nonsurgical treatment, namely, it’s an effective method for delaying disease progression or reducing disease development based on current evidence.

## Supplementary Information

**Additional file 1.** Appendix 1. Medline via OVID search strategy

## Data Availability

The datasets used and/or analyzed during the current study are available from the corresponding author on reasonable request.

## Abbreviations

CD: Core decompression
CD+BG: CD+bone graft
CD+TI: CD+tantalum rod implantation
CD+CT: CD+Cell therapy
CD+BG+CT: CD+bone graft+cell therapy
VBG: Vascularized bone graft
ONFH: Osteonecrosis of the femoral head
JPT: Joint-preserving therapy
THA: Total hip arthroplasty
HHS: Harris Hip Score
