# Supplementary material for: Efficacy of various core decompression techniques versus non-operative treatment for osteonecrosis of the femoral head: a systemic review and network meta-analysis of randomized controlled trials
Source: BMC Musculoskelet Disord. 2021 Nov 15;22:948. doi: 10.1186/s12891-021-04808-2 (PMC8594076; doi:10.1186/s12891-021-04808-2)
Supplement: Supplementary file 1 — Additional file 1. Appendix 1. Medline via OVID search strategy [file 12891_2021_4808_MOESM1_ESM.docx]

**Appendix 1.** Medline via OVID search strategy
